# Spatial and temporal aspects of visual backward masking in children and young adolescents

**DOI:** 10.3758/s13414-015-1050-y

**Published:** 2016-01-25

**Authors:** Karin S. Pilz, Marina Kunchulia, Khatuna Parkosadze, Michael H. Herzog

**Affiliations:** School of Psychology, University of Aberdeen, William Guild Building, Aberdeen, AB243FX UK; Institute of Cognitive Neurosciences, Agricultural University of Georgia, Tbilisi, Georgia; Laboratory of Psychophysics, Brain Mind Institute, School of Life Sciences, École Polytechnique Fédérale de Lausanne, Lausanne, Switzerland

**Keywords:** Development, Spatial vision, Temporal processing

## Abstract

The development of visual functions is very diverse. Some visual functions mature within the first year of life, whereas maturation for other functions extends into adolescence. The reasons for these developmental differences are largely unknown. Here, we investigated spatiotemporal processing in children (7–9 years, *n* = 15), young adolescents (11–13 years, *n* = 26), and adults (18–33 years, *n* = 24) using the shine-through visual backward-masking paradigm. We found that children had significantly longer vernier durations than either young adolescents or adults. However, children’s spatial and temporal processing of complex masks was very similar to that of young adolescents and adults. We suggest that spatiotemporal processing related to visual backward masking is already fully developed at age 7, whereas the attentional processes related to target enhancement only mature in young adolescence.

The visual system is a complex sensory system that develops more slowly than the other senses. Maturation of basic visual processes occurs within the first year of life (for a review, see Mercuri, Baranello, Romeo, Cesarini, & Ricci, 2007), whereas the development of more specific visual functions can even proceed into adolescence (Braddick & Atkinson, 2011). Different visual functions show very different developmental time courses. Orientation selectivity, for example, develops within the first 6–8 months (Candy, Skoczenski, & Norcia, 2001; Morrone & Burr, 1986), which is similar to temporal sensitivity as tested with critical flicker fusion frequency or temporal contrast sensitivity (e.g., Ellemberg, Lewis, Liu, & Maurer, 1999; Regal, 1981; for a review, see Braddick & Atkinson, 2009). However, the ability to discriminate between different motion speeds only matures during childhood (Ahmed, Lewis, Ellemberg, & Maurer, 2005; Manning, Aggen-Murphy, & Pellicano, 2012).

Studies regarding the development of spatial vision have produced mixed results, even within the same paradigm. Experiments investigating spatial contrast sensitivity have shown adult-like performance in children anywhere between 6 and 15 years of age (e.g., Arundale, 1978; Derefeldt, Lennerstrand, & Lundh, 1979; Ellemberg et al., 1999), whereas those investigating grating acuity have shown adult-like performance, for example, at age 4 (Mayer & Dobson, 1982) or age 6 (Ellemberg et al., 1999; Skoczenski & Norcia, 2002). Another paradigm that has been used to test spatial vision in children is *vernier acuity*, which is the ability to detect a small misalignment between two vertical line segments (Carkeet, Levi, & Manny, 1997; Skoczenski & Norcia, 2002; Zanker, Mohn, Weber, Zeitler-Driess, & Fahle, 1992). The misalignments that can be detected are often smaller than the resolution of retinal receptors, and therefore, vernier acuity is often termed *hyperacuity* (e.g., Levi, Klein, & Aitsebaomo, 1985; Westheimer & Hauske, 1975). One of the first studies by Zanker et al. (1992) used a preferential-looking paradigm to investigate vernier acuity in children between 2 months and 8 years of age and found adult-like vernier acuity around age 5. However, Kim et al. (2000) tested children aged between 5 and 9 years and found that vernier acuity did not mature before age 9. Skoczenski and Norcia (2002) tested infants between 1.5 and 18 months, children aged 2 to 14, and adults. Their results indicated that vernier acuity reaches adult-like values even later than age 14. Furthermore, Carkeet et al. (1997) compared children aged 3 to 12 years to adults and found vernier hyperacuity at age 3, but they suggested further improvement until adulthood. These examples show the difficulty of specifying the age at which spatial vision matures and the large differences across and even within paradigms.

Here, we investigated the visual spatial and temporal processes related to visual backward masking in children and young adolescents using the shine-through paradigm (Herzog, Fahle, & Koch, 2001; Herzog & Koch, 2001; Herzog, Koch, & Fahle, 2001). The shine-through paradigm is a very sensitive technique that combines backward masking with vernier acuity. In a first step, participants have to discriminate the offset direction of a vernier stimulus that consists of two abutting bars, of which the lower one is offset to either the left or the right. The vernier duration (VD) and the offset are determined individually so that participants can easily discriminate the offset direction. In a second step, the vernier is followed by a grating that consists of either 5 or 25 elements (Fig. 1A; Herzog, Kopmann, & Brand, 2004). For gratings with 25 elements, the vernier appears to be superimposed on the grating (*shine-through*).

**Fig. 1:**
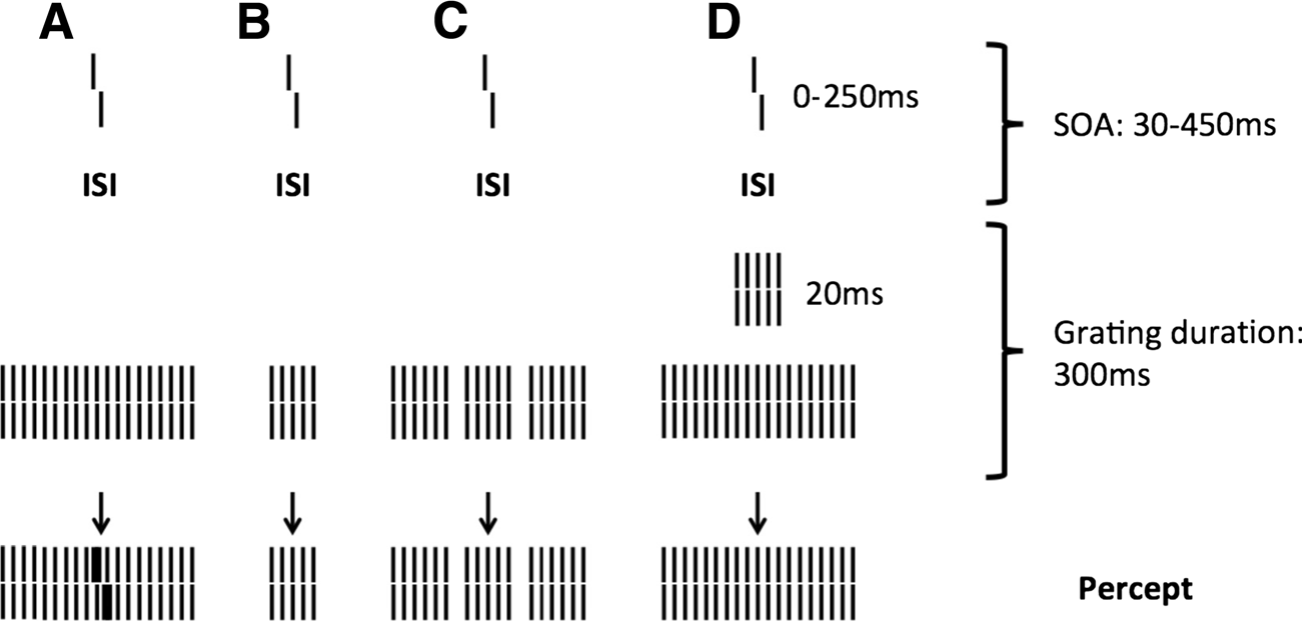
Shine-through. (A) A vernier is presented for a short time, followed by a grating with 25 aligned verniers. The target vernier appears to be superimposed on the grating. (B) For a grating with five elements, the visibility of the preceding vernier is strongly diminished. (C) The visibility of the vernier is also strongly diminished if the 25-element grating contains gaps (gap grating). (D) The visibility of the vernier is also diminished if a 5-element grating precedes the 25-element grating for a short duration of 20 ms. Only in condition A is the vernier clearly visible (*shine-through*); in all other conditions, shine-through is strongly diminished. The stimulus onset asynchrony (SOA) is determined as the sum of the vernier duration and the interstimulus interval (ISI). The bottom row shows the percept corresponding to the stimuli shown above the arrows. The depicted vernier offset is strongly exaggerated, and in the actual experiment, white bars were presented on a black background. Adapted from “Intact Figure–Ground Segmentation in Schizophrenia,” by M. H. Herzog, S. Kopmann, & A. Brand, 2004, *Psychiatry Research*, *129*, pp. 55–63. Copyright 2004 by Elsevier Ireland Ltd. Adapted with permission.

Shine-through is thought to occur because the elements of the mask are grouped as one object and the vernier is grouped as an independent element, which is therefore rendered visible (Herzog & Fahle, 2002; Herzog & Koch, 2001). For gratings with 5 elements, shine-through does not occur. This is surprising, because intuitively, the larger, 25-element grating should have more masking power, especially since it contains the 5-element grating (Fig. 1B). For this reason, the difference in masking strengths cannot be explained by retinal mechanisms alone. It has been suggested that performance reflects figure–ground segmentation: Neural responses related to the edges of the grating are strongly enhanced, whereas neural responses related to the interior elements of the grating are diminished. For small gratings, such as the 5-element grating, the strong neural responses related to the edges interfere with the responses produced by the vernier, and therefore, the visibility of the vernier is reduced (Hermens, Luksys, Gerstner, Herzog, & Ernst, 2008; Herzog, Fahle, & Koch, 2001). In a third step, the spatial and temporal processing related to backward masking are tested using inhomogeneous masks. If spatial or temporal inhomogeneities segment the 25-element grating into smaller subgratings, shine-through is decreased or even extinguished by similar mechanisms. For example, gaps inserted close to the center of the grating (*gap grating*; Fig. 1C) render the vernier invisible (Herzog & Koch, 2001; Herzog, Koch, & Fahle, 2001). Due to the gaps, the 25-element grating is segmented into two peripheral 9-element gratings and a middle 5-element grating in which, as mentioned above, shine-through does not occur. Also, briefly presenting a 5-element grating between the vernier and the 25-element grating (*5–25 grating*; Fig. 1D) dramatically deteriorates performance (Herzog, Koch, & Fahle, 2001). Even though the 5-element grating is not consciously perceived, performance commonly drops by a factor of 5, which indicates that the 5-element grating is efficiently processed by the visual system.

The shine-through paradigm has been successfully employed to test spatial and temporal processing related to backward masking in older adults (Pilz, Kunchulia, Parkosadze, & Herzog, 2015), in athletes (Overney, Blanke, & Herzog, 2008), in schizophrenia (Chkonia et al., 2010; Herzog et al., 2004), and under the influence of alcohol (Kunchulia, Pilz, & Herzog, 2012), nicotine (Kunchulia, Pilz, & Herzog, 2014), or benzodiazepines (Giersch & Herzog, 2004). Here, we tested children from age 7 to 9, young adolescents from age 11 to 13, and adults from age 18 to 33. Using the shine-through paradigm, we are able to compare spatial and temporal processing in children to that of various subgroups of the population, and thus to shed further light on the development of spatial and temporal vision. If spatial and temporal processing is not fully developed in children, we expect to find shine-through for inhomogeneous masks, which would indicate that children are unable to perceive the small spatial and temporal alterations to the grating as efficiently as young adults.

## Method

### Participants

Participants from three age groups took part in the experiment: children aged 7 to 9 years (*n* = 15, mean age = 8.1), young adolescents aged 11 to 13 (*n* = 26, mean age = 12.0), and adults aged 18 to 33 (*n* = 24, mean age = 24.5). All participants had normal or corrected-to-normal vision, as was evident from values of 0.8 or higher (equivalent to a 20/25 Snellen fraction) in the Freiburg visual acuity test (Bach, 1996). One child had to be excluded, because it was not able to perform the task. Children and adolescents were recruited from Tbilisi secondary school N100. The research was approved by the Georgian National Bioethics Committee, and the experiments were carried out in accordance with the World Medical Association Helsinki Declaration. Informed consent was obtained for all participants.

### Stimuli and apparatus

The stimuli consisted of white vertical verniers and gratings that consisted of 25 or 5 aligned verniers presented on a black background. The verniers were composed of two vertical bars that were slightly displaced in the horizontal direction, to either the left or the right, by a small gap of 1 arc min (Fig. 1). The length of one bar was 10 arc min, with a width of about 40 arc s. The single elements of the gratings were aligned verniers with a horizontal distance of about 3.33 arc min. The vernier and the central element of the grating always appeared in the middle of the screen.

Stimuli were presented on a Samsung SyncMaster 957DF CRT screen with a refresh rate of 100 Hz. The stimuli had a luminance of 100 cd/m^2^, as measured with a GretagMacbeth Eye-One Display 2 colorimeter. The background luminance of the screen was below 1 cd/m^2^, and observers were seated in a dimly lit room with a distance of 5 m from the monitor.

### Procedure

The procedure can be divided into three steps. In all three steps, observers were asked to determine the vernier offset—that is, the horizontal offset direction of the lower as compared to the upper bar (left or right). Responses were given by pressing one of two response buttons held in the left (offset to the left) and the right hand (offset to the right). In the first step, we determined the VD for the vernier presented alone—that is, without a following mask. For each observer, we determined the shortest VD for which they were able to discriminate the offset direction at a vernier offset below 40 arc s using the adaptive PEST procedure (Creelman & Taylor, 1969). The limit of 40 arc s has been used in previous publications using similar methods (e.g., Herzog, Fahle, & Koch, 2001; Herzog, Koch, & Fahle, 2001; Kunchulia et al., 2014) and is far above the normal vernier acuity for untrained observers across different age groups (Abbud & Cruz, 2002). We would like to mention that the vernier offset thresholds we obtained do not represent the true thresholds, because we stopped measuring as soon as observers reached a threshold below 40 arc s. Our main interest was to measure critical VDs. One child did not reach a critical discrimination threshold of 40 arc s, and thus was excluded from further analysis. Each observer completed as many blocks as necessary to reach a performance level of 75% correct at a vernier offset below 40 arc s. In each block of trials, the VD was constant, and only the offset was varied adaptively. Each block consisted of 80 trials. In the first block, verniers were presented for 150 ms. In the subsequent blocks, VD was reduced when the threshold for offset discrimination was below the predefined value of 40 arc s, and increased when the threshold for offset discrimination was above 40 arc s.

In the second step, the vernier was masked by a 5- or 25-element grating that were presented for 300 ms. We adaptively determined stimulus onset asynchronies (SOAs) for each grating using the PEST procedure (Creelman & Taylor, 1969). The critical SOA at which a performance level of 75% correct responses was obtained was determined using probit and maximum likelihood analysis. The SOA is defined as the difference between vernier and grating onset and is the sum of the VD and the interstimulus interval (ISI) between the vernier and the mask (SOA = VD + ISI). For each participant, we used the individual VD as determined in Step 1, and the vernier offset size was set to 71 arc s. The SOA was varied adaptively from trial to trial. We determined the critical SOA at which a performance level of 75% correct responses was obtained, using probit and maximum likelihood analysis. The starting value of the SOA was set to 200 ms. For each grating, the threshold was measured twice, and the mean of the two thresholds was taken as the critical SOA. If observers were unable to reach a threshold value of 400 ms or below, a value of 450 ms was recorded (for details, see Herzog, Fahle, & Koch, 2001).

In the third step, we tested performance on visual spatial and temporal processing using three different gratings (the standard 25-element grating [Fig. 1A], a gap grating [Fig. 1C], and a 5–25 grating [Fig. 1D]). For each observer, we used the individual SOA and VD as determined in Steps 1 and 2 to aim for the same “baseline” performance for all participants with the standard 25-element grating, to be able to explicitly assess performance changes related to the addition of temporal and spatial changes to the grating. The 25-element grating was used for baseline performance because, as we mentioned above, shine-through does not occur for 5-element gratings, and therefore it would be impossible to assess spatial and temporal aspects of backward masking using a 5-element rather than a 25-element grating. For each of the three gratings, we determined the vernier offset thresholds for a performance level of 75% correct responses using PEST. The standard 25-element grating was presented for 300 ms, as described above (Fig. 1A). The gap grating, for which two elements were removed from the standard 25-element grating, was presented for 300 ms. A gap of 250 arc s separated the central five elements from the nine elements to the left and right (Fig. 1C). For the 5–25 grating, a 25-element grating was presented for 280 ms, which was preceded by a 5-element grating presented for 20 ms, so that the duration of the combined gratings was 300 ms, the same as for the other two gratings (Fig. 1D).

It is important to note that using the shine-through paradigm, we do not investigate vernier acuity per se, which means that in the first two steps we did not vary the vernier offset, but instead determined the VD and the SOAs between the vernier and the gratings. Only in Step 3, we determined the vernier offset thresholds for inhomogeneous masks by using the predetermined VD and SOAs to investigate the effects of spatial and temporal alterations on vernier discrimination performance.

## Results

### Step 1: Vernier duration

**Fig. 2:**
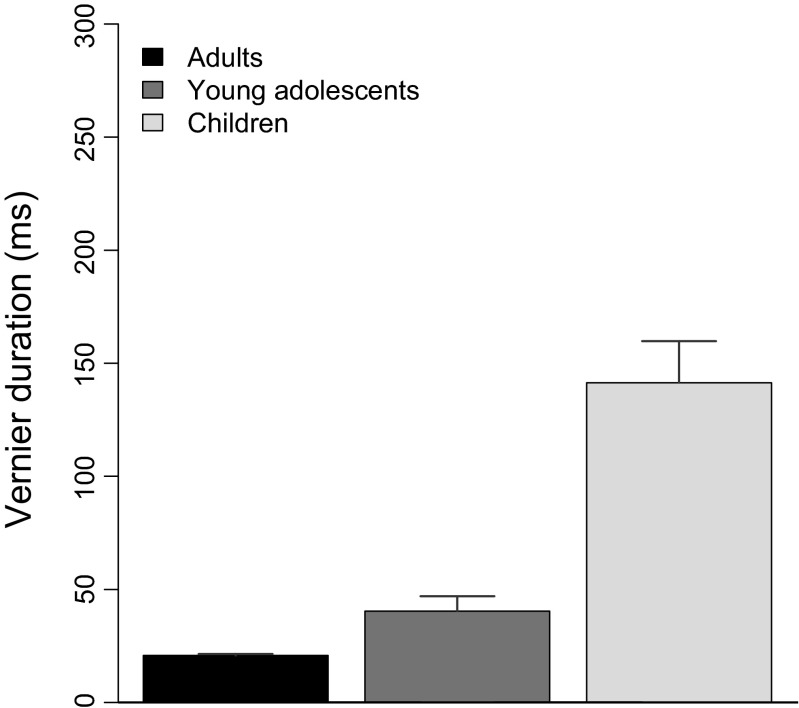
Vernier durations (VD) for adults, children, and young adolescents (Step 1). All three groups are significantly different from each other. Error bars represent standard errors of the means (*SEM*s)

An analysis of variance (ANOVA) revealed a main effect of age group [*F*(2, 67) = 34.7, *p* < .001; Fig. 2]. Post-hoc *t* tests showed significant differences between all three age groups (Table 1). Children had the longest VDs, followed by young adolescents.

**Table 1:** Two-sided *t* tests for vernier duration between adults, young adolescents and children in Step 1

	Young Adolescents	Children
Adults	*t*(25) = 2.9, *p* < .01^*^	*t*(19) = 6.2, *p* < .001^*^
Young adolescents		*t*(24) = 4.9, *p* < .001^*^

^*^ *p* < .05 indicates significant differences between age groups.

### Step 1: Vernier offset

Given that the VD was determined adaptively by varying the vernier offset, we automatically also measured thresholds for the vernier offset (see the “Procedure” section). We would like to mention that vernier offsets are not as meaningful, given that we only measured the critical VD at a vernier offset below 40 arc s. Therefore, the true thresholds for vernier offsets are likely to be lower than the measured values. An ANOVA showed a main effect of age group [*F*(2, 62) = 5.25, *p* < .01]. Post-hoc *t* tests showed significant differences between children (*M* = 32.22, *SD* = 7.9) and adults (*M* = 23.9, *SD* = 8.9) and between young adolescents (*M* = 29, *SD* = 7.7) and adults (Table 2). The difference between children and young adolescents was not significant.

**Table 2:** Two-sided *t* tests for vernier offset between adults, young adolescents and children in Step 1

	Young Adolescents	Children
Adults	*t*(45) = 2, *p* < .05^*^	*t*(32) = 3, *p* < .01^*^
Young adolescents		*t*(28) = 1.1, *p* = .2

^*^ *p* < .05 indicates significant differences between age groups.

### Step 2: Backward masking

**Fig. 3:**
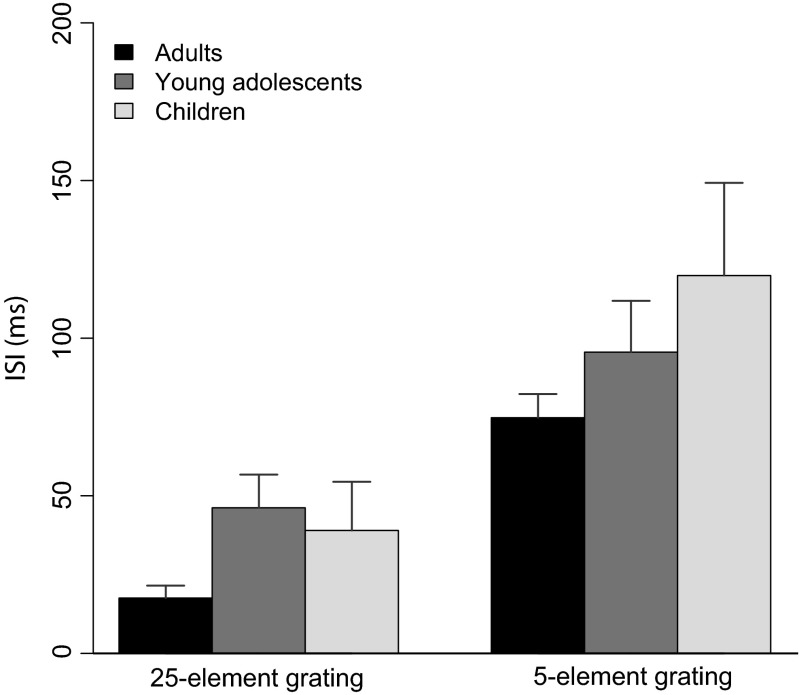
ISIs for the 25-element grating (left) and the 5-element grating (right) for adults, children, and young adolescents (Step 2). Overall, the ISIs for the 5-element grating are longer than those for the 25-element grating, but there is no difference between age groups. Error bars represent *SEM*s

A 2 (ISI) × 3 (Age Group) ANOVA revealed a main effect of ISI [*F*(1, 63) = 96.26, *p* < .001, *η*^2^ = .54], with the ISI for the 5-element grating (*M* = 96.16, *SD* = 81.73) being longer than that for the 25-element grating (*M* = 37.77, *SD* = 55.77), but no effect of age group [*F*(1, 63) = 2.5, *p* = .1, *η*^2^ = .12] and no interaction [*F*(1, 63) = 0.17, *p* = .7, *η*^2^ = .02; Fig. 3].

### Step 3: Inhomogeneous masks

**Fig. 4:**
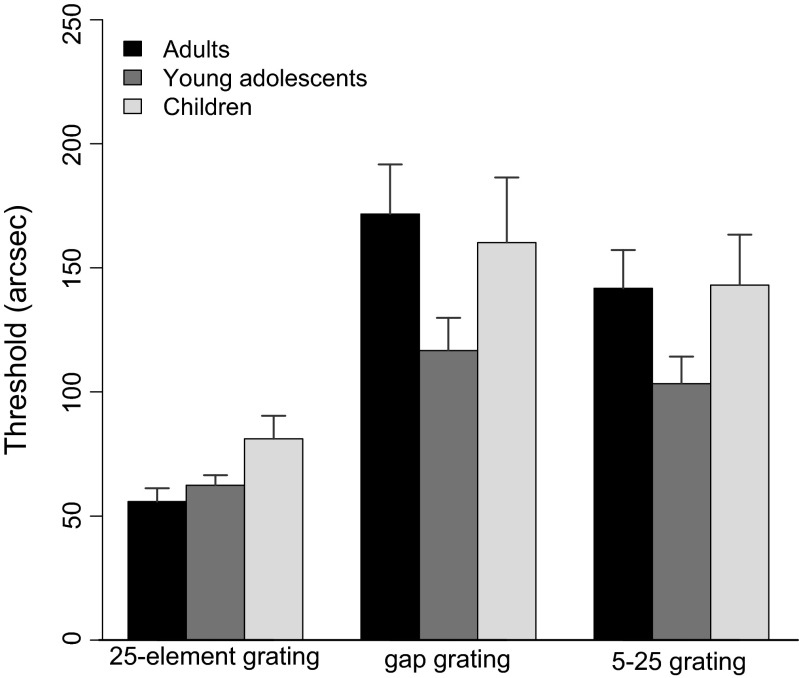
Thresholds for the 25-element, the gap, and the 5–25 grating for all participant groups (Step 3). Error bars represent *SEM*s

We used a gap grating and a 5–25 grating to investigate the effects of age on spatial and temporal processing. To compensate for individual differences, we used the individually determined VDs and ISIs from Steps 1 and 2. Due to this normalization process, we expected performance to be similar for all age groups. However, despite the normalization, children had larger thresholds than both young adolescents and adults. An ANOVA showed significant differences between the groups for the 25-element grating [*F*(2, 62) = 4.21, *p* < .05, *η*^2^ = .12], in that the thresholds for that grating were significantly higher for children than for adults [*t*(23) = 2.3, *p* < .05], but no significant difference between young adolescents and children [*t*(19) = 1.8, *p* = .08] or between young adolescents and adults [*t*(43) = 0.9, *p* = .33]. We found no significant differences between age groups for the gap [*F*(2, 62) = 2.7, *p* = .8] or the 5–25 [*F*(2, 62) = 2.5, *p* = .9; Fig. 4, Table 3] grating. To specifically test for the effects of spatial and temporal inhomogeneities, regardless of general masking performance, the thresholds for the 25-element grating were subtracted from those for the gap and 5–25 gratings, as we described above. Normalized data were significantly different from zero for both inhomogeneous gratings for all three age groups (Table 4), which indicates that spatial and temporal disturbances to the grating decrease performance.

**Table 3:** Means (*M*) and standard deviations (*SD*) for thresholds in arcsec for all three gratings and all age groups in Step 3

	Adults	Young Adolescents	Children
25 grating	55.85 (26.21)	62.42 (20.56)	97.9 (75.64)
Gap grating	171.69 (98.10)	116.67 (67.18)	172.03 (109.22)
5–25 grating	141.79 (75.29)	103.33 (55.56)	156.03 (91.67)

**Table 4:** Two-sided *t* tests for normalized data for the inhomogeneous masks in Step 3

	5–25 Grating	Gap Grating
Adults	*t*(23) = 5.1, *p* < .001^*^	*t*(23) = 5.5, *p* < .001^*^
Young adolescents	*t*(25) = 4.1, *p* < .001^*^	*t*(25) = 4.7, *p* < .001^*^
Children	*t*(14) = 3.1, *p* < .01^*^	*t*(14) = 3.3, *p* < .01^*^

^*^ *p* < .017 indicates significant differences from zero with a Bonferroni corrected alpha of .05/3.

**Fig. 5:**
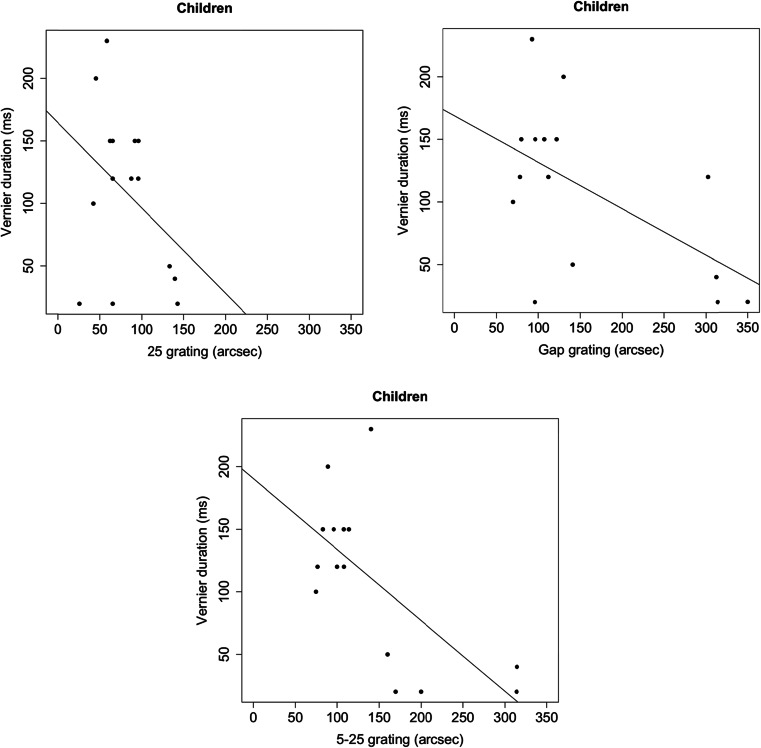
Correlations between offset thresholds for the inhomogeneous masks and vernier durations (VDs) for children. VD correlated significantly with thresholds for the gap (top right) and the 5–25 (bottom) grating, but not with thresholds for the 25-element grating (top left). We found no correlations in young adolescents or adults between VD and thresholds for inhomogeneous masks

As mentioned above, equating performance for all age groups by using individually determined VDs and ISIs was unsuccessful for children. The failed normalization in children might be related to the increased VDs, in that an increased duration could sensitize the visual system to spatial and temporal changes of the following grating. To assess the relationship between VD and performance for the gratings, we calculated Pearson correlations (Fig. 5), and found strong negative relationships between VD and performance for the gap (*r* = .57, *p* < .05) and the 5–25 (*r* = .67, *p* < .01) grating in children. Shorter VDs were related to higher grating thresholds. The relationship between VD and performance for the 25-element grating was not significant (*r* = .37, *p* = .18). Values did not correlate for young adolescents or adults, all *p*s > .5.

## Discussion

We compared spatial and temporal processing in children aged 7–9 years and young adolescents aged 11–14 to those of adults by using the shine-through visual-masking paradigm (Herzog, Fahle, & Koch, 2001; Herzog & Koch, 2001; Herzog, Fahle, & Koch, 2001).

In a first step, we measured individual vernier durations for verniers presented without a masking grating for vernier offsets below 40 arc s. VDs were significantly different between all age groups, with the longest VDs for children and the shortest for adults. In a second step, we measured ISIs for verniers that were backward-masked with a 25-element or a 5-element grating. Interestingly, there was no difference in ISIs between age groups. When we accounted for the differences in VD and ISI in a third step and tested performance for the gap (spatially inhomogeneous mask) and the 5–25 grating (temporally inhomogeneous mask), both children and young adolescents showed vernier offset thresholds similar to those of adults. These results indicate that the visual system of young adolescents is very sensitive to the briefly (20-ms) presented 5-element grating in the 5–25 condition and to the gaps in the gap condition despite increased VDs, which indicates that their visual processing is similar to that of adults.

For children, the situation is more complex. Children also showed intact spatial and temporal processing despite dramatically increased VDs, but the normalization process through which we equated individual performance for the 25-element grating by using individually determined VDs and ISIs was unsuccessful, in that children still had significantly larger thresholds than young adolescents and adults. We interpret our results as follows: Complex spatial processing (gap grating) and temporal processing (5–25 grating) of the mask are already fully developed in children as young as 7 years old. However, their much longer VDs indicate that visual processing of “weak” target elements, such as verniers with small offsets, is not fully matured in children, and even using individually determined temporal parameters, such as VD and ISI, does not alleviate this deficit for more complex stimuli, as was indicated by larger thresholds for the 25-element grating in children than in either adults or young adolescents. One could have expected that due to the failed normalization, and therefore increased thresholds for the 25-element grating in Step 3, children would have lower thresholds than adults with the gap and 5–25 gratings, but the opposite was the case.

Interestingly, we also found negative correlations between VDs and inhomogeneous masks. Children with longer VDs had lower thresholds for the gap and 5–25 gratings: That is, the more time children needed to process the unmasked vernier, the better they were at discriminating verniers masked with inhomogeneous gratings, which indicates that for children with longer VDs, the spatial and temporal alterations had little effect.

The findings from this paper relate to recent results from older adults on spatial and temporal aspects of visual backward masking (Pilz et al., 2015). Pilz et al. tested older adults (>60 years) and younger adults (<33 years) in the shine-through paradigm. Older adults were divided into two groups, depending on whether or not their VDs were comparable to those of younger adults (Step 1). Interestingly, backward masking (Step 2) and spatial and temporal aspects of visual backward masking (Step 3) were only impaired in the group of older adults who had longer VDs than younger adults. As in the results for children from this paper, the longer VDs older adults had for unmasked verniers, the better they were at discriminating verniers masked with inhomogeneous gratings. As mentioned above, shine-through is reduced by spatial or temporal alterations to the grating. Therefore, the better participants are at discriminating the vernier when it is masked with an inhomogeneous grating, the less sensitive they are to the spatial and temporal alterations. The difficulties processing those spatial and temporal alterations could potentially be explained by optical deficits. Visual acuity, however, was comparable in both groups of older adults tested by Pilz et al., which suggests that cortical rather than retinal mechanisms were responsible for the decreased performance in one group of older adults. We argue that the deficiencies in visual processing for children in this paper—and potentially also for older adults, as shown by Pilz et al.—might be based on differences in attentional mechanisms that change across age. Under normal circumstances, fine-grained visual information goes unnoticed and is likely not fully encoded in the human brain; only when it is task-relevant this information will be enhanced for further processing. The enhancement of fine-grained visual information might not be fully matured in children. Whereas visual spatiotemporal processing itself might mature at a very young age, attentional processes that are possibly linked to neuromodulatory systems develop later (Herzog, Roinishvili, Chkonia, & Brand, 2013). These two factors are usually difficult to disentangle, because observers have to pay attention to the target. However, in our paradigm, visual processing of the mask is task-irrelevant, which allows us to differentiate between visual spatiotemporal and attentional processes. Previous studies have already highlighted that attentional processes related to visual short-term memory are not fully developed at age 7 (e.g., Cowan, Morey, AuBuchon, Zwilling, & Gilchrist, 2010; Shimi, Nobre, Astle, & Scerif, 2014), and relative maturity in a variety of visual and auditory attention tasks seems to be reached around age 10 (Klenberg, Korkman, & Lahti-Nuuttila, 2001). Decreased general alertness toward the end of the experimental session might also have contributed to the failed normalization. In addition, other paradigms have shown that visual temporal processing matures at an early age: The critical flicker fusion frequency (for a review, see Braddick & Atkinson, 2009), the temporal frequency at which a flashing light or grating cannot be distinguished from a steady one, seems to mature within the first few months during childhood (Regal, 1981), and visual evoked potentials follow an adult-like flicker frequency of 55 Hz as early as 8–9 months during infancy (Apkarian, 1993; Morrone, Fiorentini, & Burr, 1996). Selective attentional processes, however, seem to mature only in young adolescence (Taylor & Khan, 2000).

To conclude, the results from our study and previous ones suggest that spatial and temporal aspects of visual backward masking are already well developed at age 7. However, deficits in target enhancement that are possibly related to differences in attention-modulating processes between age groups lead to longer VDs in children and young adolescents than in adults.

## References

[CR1] Abbud CMM, Cruz AAV (2002). Variability of vernier acuity measurements in untrained subjects of different ages. Brazilian Journal of Medical Research.

[CR2] Ahmed IJ, Lewis TL, Ellemberg D, Maurer D (2005). Discrimination of speed in 5-year-olds and adults: Are children up to speed?. Vision Research.

[CR3] Apkarian P (1993). Temporal frequency responsivity shows multiple maturational phases: State-dependent visual evoked potential luminance flicker fusion from birth to 9 months. Visual Neuroscience.

[CR4] Arundale K (1978). An investigation into the variation of human contrast sensitivity with age and ocular pathology. British Journal of Ophthalmology.

[CR5] Bach M (1996). The Freiburg visual acuity test—Automatic measurement of visual acuity. Optometry and Vision Science.

[CR6] Braddick O, Atkinson J (2009). Infants’ sensitivity to motion and temporal change. Optometry and Vision Science.

[CR7] Braddick O, Atkinson J (2011). Development of human visual function. Vision Research.

[CR8] Candy TR, Skoczenski AM, Norcia AM (2001). Normalization models applied to orientation masking in the human infant. Journal of Neuroscience.

[CR9] Carkeet A, Levi DM, Manny RE (1997). Development of vernier acuity in childhood. Optometry and Vision Science.

[CR10] Chkonia E, Roinishvili M, Makhatadze N, Tsverava L, Stroux A, Neumann K, Brand A (2010). The shine-through masking paradigm is a potential endophenotype of schizophrenia. PLoS ONE.

[CR11] Cowan N, Morey CC, AuBuchon AM, Zwilling CE, Gilchrist AL (2010). Seven-year-olds allocate attention like adults unless working memory is overloaded. Developmental Science.

[CR12] Creelman CD, Taylor MM (1969). Some pitfalls in adaptive testing: comments on “temporal integration and periodicity pitch.”. Journal of the Acoustical Society of America.

[CR13] Derefeldt G, Lennerstrand G, Lundh B (1979). Age variations in normal human contrast sensitivity. Acta Ophthalmologica.

[CR14] Ellemberg D, Lewis TL, Liu CH, Maurer D (1999). Development of spatial and temporal vision during childhood. Vision Research.

[CR15] Giersch A, Herzog MH (2004). Lorazepam strongly prolongs visual information processing. Neuropsychopharmacology.

[CR16] Hermens F, Luksys G, Gerstner W, Herzog MH, Ernst U (2008). Modeling spatial and temporal aspects of visual backward masking. Psychological Review.

[CR17] Herzog, M. H., & Fahle, M. (2002). Effects of grouping in contextual modulation. *Nature, 415*, 433–436. 10.1038/415433a 11807555

[CR18] Herzog MH, Fahle M, Koch C (2001). Spatial aspects of object formation revealed by a new illusion, shine-through. Vision Research.

[CR19] Herzog MH, Koch C (2001). Seeing properties of an invisible object: Feature inheritance and shine-through. Proceedings of the National Academy of Science.

[CR20] Herzog MH, Koch C, Fahle M (2001). Shine-through: Temporal aspects. Vision Research.

[CR21] Herzog MH, Kopmann S, Brand A (2004). Intact figure–ground segmentation in schizophrenia. Psychiatry Research.

[CR22] Herzog MH, Roinishvili M, Chkonia E, Brand A (2013). Schizophrenia and visual backward masking: a general deficit of target enhancement. Frontiers in Psychology.

[CR23] Kim E, Enoch JM, Fang MS, Lakshminarayanan V, Kono M, Strada E, Srinivasan R (2000). Performance on the three-point vernier alignment or acuity test as a function of age: Measurement extended to ages 5 to 9 years. Optometry and Vision Science.

[CR24] Klenberg L, Korkman M, Lahti-Nuuttila P (2001). Differential development of attention and executive functions in 3- to 12-year-old Finnish children. Developmental Neuropsychology.

[CR25] Kunchulia M, Pilz KS, Herzog MH (2012). How alcohol intake affects visual temporal processing. Vision Research.

[CR26] Kunchulia M, Pilz KS, Herzog MH (2014). Small effects of smoking on visual spatiotemporal processing. Scientific Reports.

[CR27] Levi DM, Klein SA, Aitsebaomo AP (1985). Vernier acuity, crowding and cortical magnification. Vision Research.

[CR28] Mayer DL, Dobson V (1982). Visual acuity development in infants and young children, as assessed by operant preferential looking. Vision Research.

[CR29] Manning C, Aggen-Murphy D, Pellicano E (2012). The development of speed discrimination abilities. Vision Research.

[CR30] Mercuri E, Baranello G, Romeo DMM, Cesarini L, Ricci D (2007). The development of vision. Early Human Development.

[CR31] Morrone MC, Burr DC (1986). Evidence for the existence and development of visual inhibition in humans. Nature.

[CR32] Morrone MC, Fiorentini A, Burr DC (1996). Development of the temporal properties of visual evoked potentials to luminance and colour contrast in infants. Vision Research.

[CR33] Overney LS, Blanke O, Herzog MH (2008). Enhanced temporal but not attentional processing in expert tennis players. PLoS ONE.

[CR34] Pilz KS, Kunchulia M, Parkosadze K, Herzog MH (2015). Ageing and visual spatiotemporal processing. Experimental Brain Research.

[CR35] Regal DM (1981). Development of critical flicker frequency in human infants. Vision Research.

[CR36] Shimi A, Nobre AC, Astle D, Scerif G (2014). Orienting attention within visual short-term memory: Development and mechanisms. Child Development.

[CR37] Skoczenski AM, Norcia AM (2002). Late maturation of visual hyperacuity. Psychological Science.

[CR38] Taylor MJ, Khan SC (2000). Top-down modulation of early selective attention processes in childhood. International Journal of Psychophysiology.

[CR39] Westheimer G, Hauske G (1975). Temporal and spatial interference with vernier acuity. Vision Research.

[CR40] Zanker J, Mohn G, Weber U, Zeitler-Driess K, Fahle M (1992). The development of vernier acuity in human infants. Vision Research.

